# Crystal structure of (nitrato-κ*O*)bis­(1,10′-phenanthroline-κ^2^ *N*,*N*′)copper(II) nitrate gallic acid monosolvate monohydrate

**DOI:** 10.1107/S2056989016016066

**Published:** 2016-10-14

**Authors:** Fwu Ming Shen, Shie Fu Lush

**Affiliations:** a Department of Biotechnology, Yuanpei University, No.306, Yuanpei St., HsinChu, 30015, Taiwan; b Department of Medical Laboratory Science and Biotechnology, Yuanpei University, HsinChu, 30015, Taiwan

**Keywords:** crystal structure, trigonal–bipyramidal coordination, Cu complex, phenanthroline ligand

## Abstract

The title compound, [Cu(NO_3_)(C_12_H_8_N_2_)_2_]NO_3_·C_7_H_6_O_5_·H_2_O, consists of a mononuclear complex cation with the central Cu^II^ atom in a distorted trigonal–bipyramidal coordination sphere. Two N atoms of two 1,10-phenanthroline ligands occupy the axial sites, and the remaining N atoms of the two ligands, as well as one nitrate O atom the equatorial positions. One mol­ecule each of gallic acid and water are present in the crystal as solvent mol­ecules that do not coordinate to the Cu^II^ cation, just as the nitrate counter-anion. In the crystal, inter­molecular O—H⋯O hydrogen bonds, as well as C—H⋯O inter­actions and π–π ring stacking between benzene and pyridine rings [centroid-to-centroid distances = 3.471 (2), 3.559 (2) and 3.790 (2) Å], link the mol­ecules into a three-dimensional network structure.

## Abstract 2

The coordination sphere of the Cu^II^ atom in the title compound is trigonal–bipyramidal, with two N atoms of two 1,10-phenanthroline ligands occupying the axial sites, and the remaining N atoms of the ligands, as well as one O atom of a nitrate anion occupying the equatorial positions.

## Chemical context   

Numerous metal complexes with polypyridine-containing ligands have been reported. One such ligand is 1,10′-phenanthroline (phen). For transition metal complexes of phen, excellent photoelectrical capabilities have been reported (Dumur *et al.*, 2009 ▸). Moreover, [Cu(phen)] complexes are applied in breaking the DNA chain (Selvakumar *et al.*, 2006 ▸).
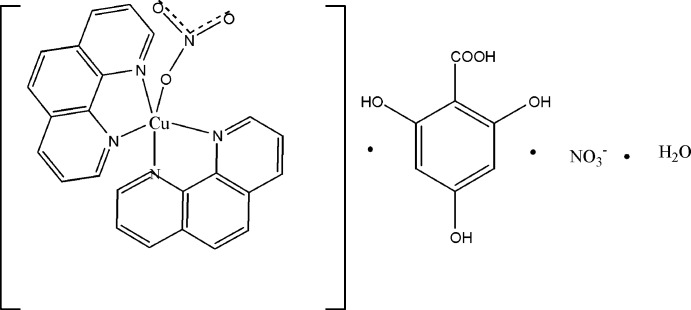


The nitrate ligand shows a great variation in its coordination behaviour. A number of coordination modes have been observed upon inter­action with a metal ion (Wyllie *et al.*, 2007 ▸). For compounds with [Cu(phen)NO_3_] moieties, non-bridging coordination modes of nitrate ligands range from monodentate (κ^1^) (Seidel *et al.*, 2011 ▸), asymmetric bidentate (κ^2^) (Chen *et al.*, 2005 ▸) to symmetric bidentate (κ^2^) (Ovens *et al.*, 2010 ▸).

In a project to combine phen and nitrate ligands with gallic acid as an additional co-ligand for coordination to a Cu^II^ atom, we obtained the title compound, [Cu(NO_3_)(C_12_H_8_N_2_)_2_]NO_3_·C_7_H_6_O_5_·H_2_O. However, as revealed by single crystal X-ray diffraction analysis, gallic acid does not coordinate to the metal but is incorporated as a solvent mol­ecule.

## Structural commentary   

**Figure 1:**
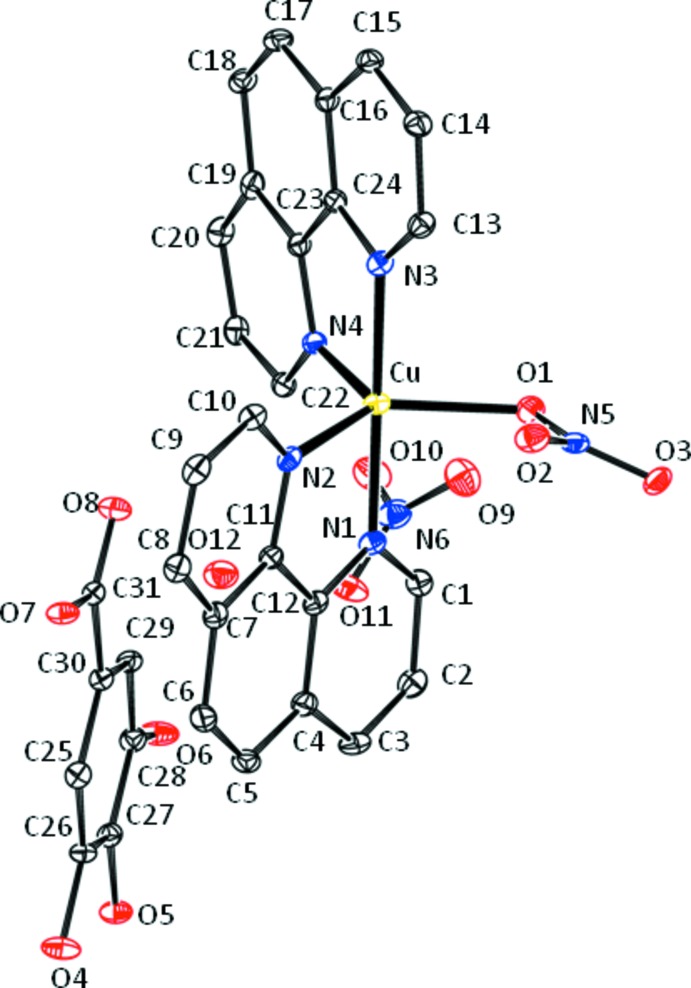
The asymmetric unit of the title compound, showing the atom-numbering scheme. Displacement ellipsoids are drawn at the 50% probability level. H atoms have been omitted for clarity.

The coordination sphere around copper in the complex cation, [Cu(NO_3_)(C_12_H_8_N_2_)_2_]^+^, comprises one oxygen atom (O1) of one nitrate anion and four nitro­gen atoms (N1, N2, N3, N4) of two *N,N*’-chelating phen ligands (Fig. 1 ▸, Table 1 ▸). The conformation of the resulting N_4_O coordination set is distorted trigonal–bipyramidal, as revealed by the structural parameter τ_5_ (Addison *et al.*, 1984 ▸), which is defined as τ = (β − α) /60 where β and α are the two greatest angles of the coordinated atom. For a perfect square–pyramidal coordination, τ is 0, and for perfect trigonal–bipyramidal coordination, τ becomes 1.0. In the title compound, the largest angles are β = 178.59 (10)° for N1—Cu—N3, and α = 132.61 (9) ° for O1—Cu—N2. Thus, τ is 0.76, indicating a considerable distortion. Each phen ligand provides an equatorial (N2, N4) and an axial (N1, N3) nitro­gen donor atom, forming five-membered chelate rings. The fifth coordination site is occupied by an equatorial oxygen atom (O1) from one of the nitrate anions. The axial distances are shorter than the equatorial distances; relevant bond lengths and angles are collated in Table 1 ▸. The dihedral angle between two phen planes around the metal cation is 64.45 (7)°.

There is an additional inter­action of the copper cation with atom O2 of the nitrate ligand. This inter­action is rather weak [2.782 (2) Å], and the result of a bond-valence-sum calculation (Brown & Altermatt, 1985 ▸) reveals a valence unit of 0.047 for O2, which is lower than the limit of 0.06 for a cation–donor contact to be considered as a weak bonding inter­action (Liebau, 2000 ▸).

## Supra­molecular features   

**Figure 2:**
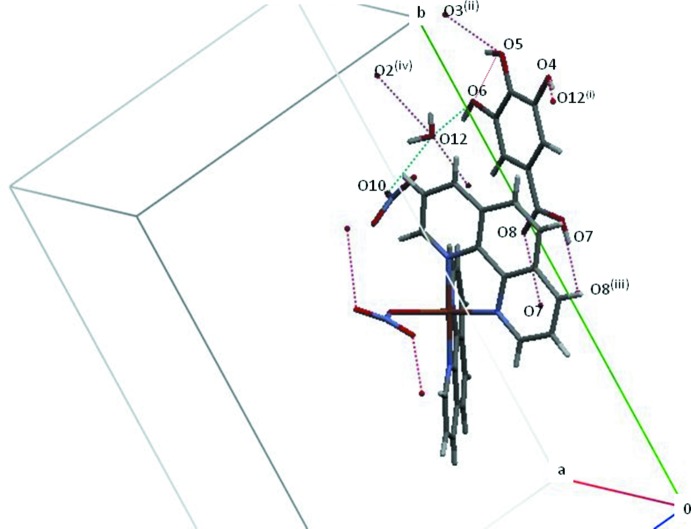
Parts of the crystal structure of the title compound sustained by O—H⋯O hydrogen bonds (dotted lines).

**Figure 3:**
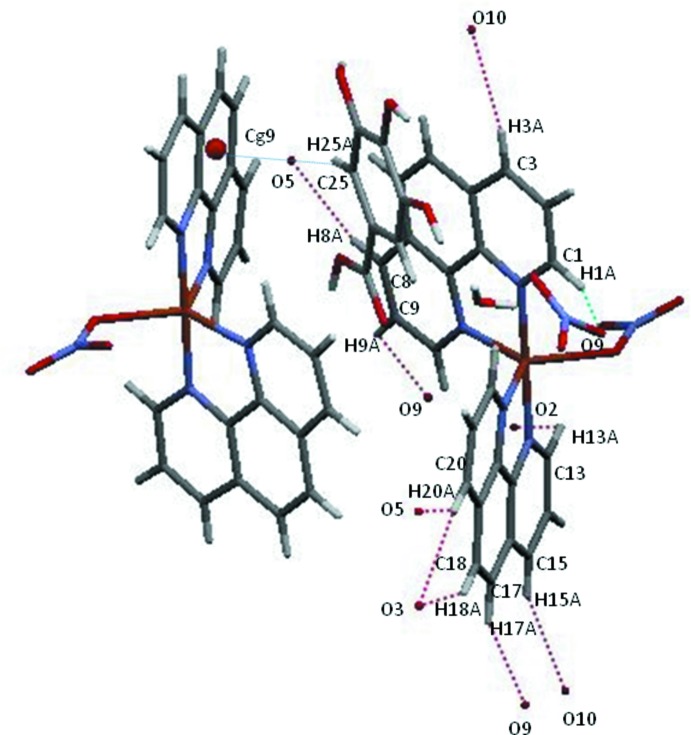
Inter­molecular O—H⋯O and C—H⋯O hydrogen bonds (dotted lines), as well as C—H⋯π inter­actions in the crystal structure of the title compound.

As already noted in Section 1, gallic acid does not coordinate to the metal but is involved in numerous hydrogen-bonding inter­actions, including one intra­molecular hydrogen bond between one of the hy­droxy groups (O5) and neighbouring O6. In the crystal, inter­molecular O—H⋯O bonds between the other OH functions of gallic acid as well as of the water solvent mol­ecule are present. The latter also is hydrogen-bonded to O2 of the coordinating nitrate group and to O10 of the non-coordinating nitrate counter-anion (Table 2 ▸), establishing a three-dimensional network that is consolidated by further C—H⋯O hydrogen-bonding inter­actions (Table 2 ▸, Figs. 2 ▸ and 3 ▸). In addition to these classical and non-classical hydrogen-bonding inter­actions, π–π ring stacking between benzene and pyridine rings with centroid-to-centroid distances in the range 3.471 (2)–3.992 (2)Å is observed, the shortest distance being between *Cg*8(C4–C7/C11–C12) and its symmetry-related counterpart [symmetry code: 1 − *x*, 1 − *y*, −*z*]. Finally, C—H⋯π inter­actions (Table 2 ▸, Fig. 3 ▸) are also present.

## Synthesis and crystallization   

The reagents Cu(NO_3_)_2_·6H_2_O, gallic acid and phen were used as commercially received. A warm solution of phen (0.180 g, 1 mmol) and gallic acid (0.170 g, 1mmol) in a ethanol/water mixture (20 ml) was added to a solution of Cu(NO_3_)_2_·6H_2_O (0.296 g, 1 mmol) in the same solvent (20 ml). The mixture was refluxed for 1 h and the green solution filtered. Upon slow evaporation of the solvent at room temperature, a green crystalline solid appeared several weeks later and was separ­ated by filtration. Elemental analysis: calculated (%) C_31_H_24_CuN_6_O_12_: C 50.58, H 3.29, N 11.42; found C 50.62, H 3.39, N 11.50.

## Refinement details   

Crystal data, data collection and structure refinement details are summarized in Table 3 ▸. C-bound H atoms were positioned geometrically with C—H = 0.95 Å and were refined using a riding model with *U*_iso_(H) = 1.2*U*_eq_(C) All O-bound H atoms were located in a difference Fourier map and were refined with distances constraints of O—H = 0.82 Å and *U*_iso_(H) = 1.5*U*_eq_(O).

## Supplementary Material

Crystal structure: contains datablock(s) global, I. DOI: 10.1107/S2056989016016066/wm5328sup1.cif

Structure factors: contains datablock(s) I. DOI: 10.1107/S2056989016016066/wm5328Isup2.hkl

CCDC reference: 885877

Additional supporting information: crystallographic information; 3D view; checkCIF report

## Figures and Tables

**Table 1:** Selected geometric parameters (Å, °)

Cu—O1	2.114 (2)	Cu—N2	2.082 (3)
Cu—O2	2.782 (2)	Cu—N3	1.980 (3)
Cu—N1	1.974 (3)	Cu—N4	2.086 (2)
			
O1—Cu—O2	50.52 (8)	N2—Cu—N4	121.57 (10)
O1—Cu—N1	86.42 (10)	N3—Cu—N4	81.84 (10)
O1—Cu—N2	132.61 (9)	Cu—O1—N5	109.30 (19)
O1—Cu—N3	93.74 (10)	Cu—O2—N5	78.26 (15)
O1—Cu—N4	105.34 (9)	Cu—N1—C1	127.4 (2)
O2—Cu—N1	93.06 (8)	Cu—N1—C12	114.0 (2)
O2—Cu—N2	84.29 (8)	Cu—N2—C11	110.5 (2)
O2—Cu—N3	88.12 (8)	Cu—N2—C10	132.0 (2)
O2—Cu—N4	153.32 (9)	Cu—N3—C24	113.6 (2)
N1—Cu—N2	82.09 (11)	Cu—N3—C13	127.7 (2)
N1—Cu—N3	178.59 (10)	Cu—N4—C22	131.2 (2)
N1—Cu—N4	96.77 (10)	Cu—N4—C23	110.3 (2)
N2—Cu—N3	98.81 (11)		

**Table 2:** Hydrogen-bond geometry (Å, °) *Cg*9 is the centroid of the C16–C19/C23/C24 ring.

*D*—H⋯*A*	*D*—H	H⋯*A*	*D*⋯*A*	*D*—H⋯*A*
O4—H4*A*⋯O12^i^	0.82	1.92	2.730 (3)	167
O5—H5*B*⋯O6	0.82	2.13	2.624 (3)	119
O5—H5*B*⋯O3^ii^	0.82	2.16	2.859 (3)	143
O6—H6*B*⋯O12	0.82	1.94	2.682 (3)	150
O7—H7*A*⋯O8^iii^	0.82	1.84	2.648 (3)	170
O12—H12*A*⋯O2^iv^	0.82	2.55	2.960 (3)	112
O12—H12*B*⋯O10	0.82	2.13	2.708 (4)	128
C1—H1*A*⋯O9	0.95	2.56	3.338 (4)	139
C3—H3*A*⋯O10^i^	0.95	2.48	3.395 (4)	161
C8—H8*A*⋯O5^v^	0.95	2.56	3.482 (4)	165
C9—H9*A*⋯O9^vi^	0.95	2.52	3.166 (4)	125
C13—H13*A*⋯O2	0.95	2.59	3.254 (4)	128
C15—H15*A*⋯O10^vii^	0.95	2.56	3.457 (4)	158
C17—H17*A*⋯O9^vii^	0.95	2.49	3.340 (5)	150
C18—H18*A*⋯O3^viii^	0.95	2.55	3.363 (4)	144
C20—H20*A*⋯O3^viii^	0.95	2.44	3.282 (4)	148
C20—H20*A*⋯O5^ix^	0.95	2.55	3.346 (4)	141
C25—H25*A*⋯*Cg*9^iii^	0.95	2.95	3.680 (3)	135

Symmetry codes: (i) 
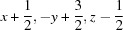
; (ii) 
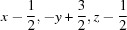
; (iii) 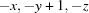; (iv) 
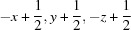
; (v) 
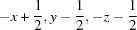
; (vi) 
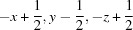
; (vii) 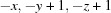; (viii) 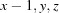; (ix) 
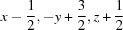
.

**Table 3:** Experimental details

Crystal data	
Chemical formula	[Cu(NO_3_)(C_12_H_8_N_4_)_2_]NO_3_·C_7_H_6_O_5_·H_2_O
*M*_r_	736.11
Crystal system, space group	Monoclinic, *P*2_1_/*n*
Temperature (K)	110
*a*, *b*, *c* (Å)	11.0235 (4), 20.5399 (9), 12.9222 (5)
β (°)	93.250 (3)
*V* (Å^3^)	2921.2 (2)
*Z*	4
Radiation type	Mo *K*α
μ (mm^−1^)	0.83
Crystal size (mm)	0.48 × 0.42 × 0.17

Data collection	
Diffractometer	Oxford Diffraction Gemini-S CCD detector
Absorption correction	Multi-scan (*CrysAlis PRO*; Oxford Diffraction, 2009 ▸)
*T*_min_, *T*_max_	0.513, 1.000
No. of measured, independent and observed [*I* > 2σ(*I*)] reflections	11305, 5136, 4223
*R*_int_	0.060
(sin θ/λ)_max_ (Å^−1^)	0.595

Refinement	
*R*[*F* ^2^ > 2σ(*F* ^2^)], *wR*(*F* ^2^), *S*	0.051, 0.137, 1.10
No. of reflections	5136
No. of parameters	451
H-atom treatment	H-atom parameters constrained
Δρ_max_, Δρ_min_ (e Å^−3^)	1.03, −0.70

Computer programs: *CrysAlis CCD* and *CrysAlis RED* (Oxford Diffraction, 2009 ▸), *SHELXS97* and *SHELXL97* (Sheldrick, 2008 ▸) *PLATON* (Spek, 2009 ▸).

## supplementary crystallographic information

### Crystal data

**Table:**

[Cu(NO_3_)(C_12_H_8_N_4_)_2_]NO_3_·C_7_H_6_O_5_·H_2_O	*F*(000) = 1508
*M**_r_* = 736.11	*D*_x_ = 1.674 Mg m^−^^3^
Monoclinic, *P*2_1_/*n*	Mo *K*α radiation, λ = 0.71073 Å
Hall symbol: -P 2yn	Cell parameters from 3946 reflections
*a* = 11.0235 (4) Å	θ = 3.0–29.2°
*b* = 20.5399 (9) Å	µ = 0.83 mm^−^^1^
*c* = 12.9222 (5) Å	*T* = 110 K
β = 93.250 (3)°	Parallelepiped, green
*V* = 2921.2 (2) Å^3^	0.48 × 0.42 × 0.17 mm
*Z* = 4	

### Data collection

**Table:**

Oxford Diffraction Gemini-S CCD detector diffractometer	4223 reflections with *I* > 2σ(*I*)
ω scans	*R*_int_ = 0.060
Absorption correction: multi-scan (*CrysAlis PRO*; Oxford Diffraction, 2009)	θ_max_ = 25.0°, θ_min_ = 3.1°
*T*_min_ = 0.513, *T*_max_ = 1.000	*h* = −12→13
11305 measured reflections	*k* = −24→22
5136 independent reflections	*l* = −10→15

### Refinement

**Table:**

Refinement on *F*^2^	Primary atom site location: structure-invariant direct methods
Least-squares matrix: full	Secondary atom site location: difference Fourier map
*R*[*F*^2^ > 2σ(*F*^2^)] = 0.051	Hydrogen site location: inferred from neighbouring sites
*wR*(*F*^2^) = 0.137	H-atom parameters constrained
*S* = 1.10	*w* = 1/[σ^2^(*F*_o_^2^) + (0.070*P*)^2^ + 0.842*P*] where *P* = (*F*_o_^2^ + 2*F*_c_^2^)/3
5136 reflections	(Δ/σ)_max_ = 0.001
451 parameters	Δρ_max_ = 1.03 e Å^−^^3^
0 restraints	Δρ_min_ = −0.70 e Å^−^^3^

### Special details

**Table:**

Geometry. Bond distances, angles etc. have been calculated using the rounded fractional coordinates. All su's are estimated from the variances of the (full) variance-covariance matrix. The cell esds are taken into account in the estimation of distances, angles and torsion angles
Refinement. Refinement on F^2^ for ALL reflections except those flagged by the user for potential systematic errors. Weighted R-factors wR and all goodnesses of fit S are based on F^2^, conventional R-factors R are based on F, with F set to zero for negative F^2^. The observed criterion of F^2^ > 2sigma(F^2^) is used only for calculating -R-factor-obs etc. and is not relevant to the choice of reflections for refinement. R-factors based on F^2^ are statistically about twice as large as those based on F, and R-factors based on ALL data will be even larger.

### Fractional atomic coordinates and isotropic or equivalent isotropic displacement parameters (Å^2^)

**Table:**

	*x*	*y*	*z*	*U*_iso_*/*U*_eq_	
Cu	0.24268 (3)	0.49115 (2)	0.26191 (3)	0.0175 (1)	
O1	0.36408 (19)	0.53000 (12)	0.37852 (18)	0.0248 (8)	
O2	0.4785 (2)	0.45309 (12)	0.32175 (19)	0.0294 (8)	
O3	0.55944 (19)	0.53508 (12)	0.40721 (19)	0.0259 (8)	
N1	0.2982 (2)	0.56158 (13)	0.1721 (2)	0.0178 (8)	
N2	0.2690 (2)	0.43530 (13)	0.1306 (2)	0.0181 (8)	
N3	0.1835 (2)	0.42183 (13)	0.3528 (2)	0.0179 (8)	
N4	0.0738 (2)	0.53148 (13)	0.2892 (2)	0.0163 (8)	
N5	0.4694 (2)	0.50542 (14)	0.3699 (2)	0.0202 (9)	
C1	0.3180 (3)	0.62347 (16)	0.1976 (3)	0.0197 (10)	
C2	0.3603 (3)	0.66881 (17)	0.1278 (3)	0.0242 (11)	
C3	0.3816 (3)	0.64980 (16)	0.0288 (3)	0.0228 (11)	
C4	0.3626 (3)	0.58449 (17)	−0.0009 (3)	0.0213 (10)	
C5	0.3880 (3)	0.55843 (18)	−0.1007 (3)	0.0227 (10)	
C6	0.3723 (3)	0.49417 (17)	−0.1218 (3)	0.0236 (10)	
C7	0.3316 (3)	0.44980 (17)	−0.0461 (3)	0.0203 (10)	
C8	0.3203 (3)	0.38203 (16)	−0.0619 (3)	0.0223 (10)	
C9	0.2854 (3)	0.34349 (17)	0.0174 (3)	0.0230 (11)	
C10	0.2595 (3)	0.37185 (16)	0.1129 (3)	0.0204 (10)	
C11	0.3053 (3)	0.47373 (16)	0.0517 (2)	0.0179 (10)	
C12	0.3211 (3)	0.54177 (16)	0.0736 (2)	0.0172 (9)	
C13	0.2448 (3)	0.37017 (16)	0.3906 (3)	0.0211 (10)	
C14	0.1916 (3)	0.32439 (16)	0.4533 (3)	0.0213 (10)	
C15	0.0723 (3)	0.33119 (16)	0.4773 (2)	0.0207 (10)	
C16	0.0063 (3)	0.38625 (16)	0.4397 (2)	0.0189 (10)	
C17	−0.1173 (3)	0.39922 (17)	0.4628 (3)	0.0207 (10)	
C18	−0.1745 (3)	0.45470 (16)	0.4287 (3)	0.0214 (10)	
C19	−0.1132 (3)	0.50163 (16)	0.3676 (3)	0.0199 (10)	
C20	−0.1650 (3)	0.56087 (16)	0.3325 (3)	0.0214 (10)	
C21	−0.0978 (3)	0.60333 (16)	0.2783 (3)	0.0208 (10)	
C22	0.0222 (3)	0.58736 (16)	0.2575 (2)	0.0183 (9)	
C23	0.0076 (3)	0.48935 (16)	0.3434 (2)	0.0170 (9)	
C24	0.0661 (3)	0.43054 (16)	0.3790 (2)	0.0171 (9)	
O4	0.1791 (2)	0.74367 (11)	−0.30357 (18)	0.0274 (8)	
O5	0.0913 (2)	0.84202 (11)	−0.19158 (18)	0.0219 (7)	
O6	−0.0195 (2)	0.82192 (11)	−0.02000 (19)	0.0292 (8)	
O7	0.07418 (19)	0.53917 (11)	−0.10861 (17)	0.0210 (7)	
O8	−0.0154 (2)	0.57532 (11)	0.03158 (18)	0.0237 (7)	
C25	0.1118 (3)	0.66503 (16)	−0.1797 (2)	0.0185 (10)	
C26	0.1243 (3)	0.72826 (16)	−0.2142 (2)	0.0178 (10)	
C27	0.0777 (3)	0.77938 (16)	−0.1584 (2)	0.0189 (10)	
C28	0.0202 (3)	0.76750 (16)	−0.0667 (3)	0.0196 (10)	
C29	0.0087 (3)	0.70440 (16)	−0.0309 (3)	0.0185 (10)	
C30	0.0536 (3)	0.65305 (16)	−0.0874 (2)	0.0180 (10)	
C31	0.0348 (3)	0.58604 (16)	−0.0499 (2)	0.0174 (9)	
O9	0.1588 (2)	0.71367 (13)	0.3566 (2)	0.0379 (9)	
O10	−0.0026 (2)	0.77280 (13)	0.3243 (2)	0.0345 (8)	
O11	0.1125 (2)	0.74807 (13)	0.2005 (2)	0.0373 (9)	
N6	0.0913 (3)	0.74467 (14)	0.2930 (3)	0.0298 (10)	
O12	−0.1235 (2)	0.83188 (13)	0.16200 (19)	0.0307 (8)	
H1A	0.30290	0.63730	0.26580	0.0240*	
H2A	0.37430	0.71260	0.14870	0.0290*	
H3A	0.40910	0.68060	−0.01950	0.0270*	
H5A	0.41600	0.58660	−0.15260	0.0270*	
H6A	0.38890	0.47830	−0.18860	0.0280*	
H8A	0.33680	0.36340	−0.12690	0.0270*	
H9A	0.27870	0.29770	0.00810	0.0280*	
H10A	0.23430	0.34440	0.16690	0.0250*	
H13A	0.32710	0.36450	0.37420	0.0250*	
H14A	0.23780	0.28840	0.47960	0.0260*	
H15A	0.03490	0.29950	0.51850	0.0250*	
H17A	−0.15980	0.36870	0.50250	0.0250*	
H18A	−0.25600	0.46250	0.44560	0.0260*	
H20A	−0.24640	0.57120	0.34660	0.0260*	
H21A	−0.13200	0.64350	0.25480	0.0250*	
H22A	0.06820	0.61730	0.21960	0.0220*	
H4A	0.23120	0.71620	−0.31320	0.0410*	
H5B	0.06220	0.86560	−0.14830	0.0330*	
H6B	−0.04560	0.81060	0.03540	0.0440*	
H7A	0.04780	0.50450	−0.08780	0.0320*	
H25A	0.14240	0.62980	−0.21820	0.0220*	
H29A	−0.02960	0.69630	0.03180	0.0220*	
H12A	−0.14580	0.86860	0.14490	0.0460*	
H12B	−0.08230	0.84000	0.21530	0.0460*	

### Atomic displacement parameters (Å^2^)

**Table:**

	*U*^11^	*U*^22^	*U*^33^	*U*^12^	*U*^13^	*U*^23^
Cu	0.0172 (2)	0.0165 (3)	0.0193 (2)	−0.0003 (2)	0.0049 (2)	0.0015 (2)
O1	0.0177 (12)	0.0316 (14)	0.0253 (13)	0.0014 (10)	0.0025 (10)	0.0024 (11)
O2	0.0326 (13)	0.0278 (15)	0.0287 (14)	−0.0032 (11)	0.0110 (11)	−0.0052 (12)
O3	0.0158 (11)	0.0293 (14)	0.0327 (14)	−0.0052 (10)	0.0011 (10)	0.0024 (11)
N1	0.0161 (13)	0.0159 (15)	0.0218 (14)	0.0007 (10)	0.0050 (11)	−0.0011 (12)
N2	0.0127 (12)	0.0189 (15)	0.0228 (14)	−0.0011 (10)	0.0008 (11)	0.0003 (12)
N3	0.0164 (13)	0.0162 (15)	0.0216 (14)	0.0008 (11)	0.0043 (11)	−0.0025 (12)
N4	0.0173 (13)	0.0160 (15)	0.0158 (13)	−0.0020 (11)	0.0028 (11)	−0.0006 (11)
N5	0.0192 (15)	0.0237 (17)	0.0184 (14)	−0.0013 (12)	0.0071 (12)	0.0038 (13)
C1	0.0190 (16)	0.0207 (19)	0.0197 (17)	0.0029 (13)	0.0029 (13)	−0.0019 (14)
C2	0.0231 (17)	0.0176 (19)	0.032 (2)	−0.0002 (14)	0.0013 (15)	−0.0006 (16)
C3	0.0222 (17)	0.0176 (19)	0.0292 (19)	−0.0009 (13)	0.0073 (14)	0.0064 (15)
C4	0.0140 (15)	0.027 (2)	0.0230 (18)	0.0010 (13)	0.0015 (13)	0.0049 (15)
C5	0.0174 (16)	0.031 (2)	0.0202 (17)	0.0012 (14)	0.0056 (13)	0.0045 (16)
C6	0.0181 (17)	0.034 (2)	0.0189 (17)	0.0018 (14)	0.0032 (14)	−0.0011 (15)
C7	0.0132 (15)	0.027 (2)	0.0208 (17)	0.0019 (13)	0.0024 (13)	−0.0027 (15)
C8	0.0193 (16)	0.024 (2)	0.0239 (18)	0.0012 (13)	0.0033 (14)	−0.0054 (15)
C9	0.0197 (16)	0.0184 (19)	0.031 (2)	0.0009 (13)	0.0024 (14)	−0.0064 (15)
C10	0.0179 (16)	0.0173 (18)	0.0263 (19)	0.0005 (13)	0.0030 (14)	0.0004 (14)
C11	0.0124 (15)	0.0226 (19)	0.0188 (16)	0.0002 (13)	0.0023 (12)	0.0010 (14)
C12	0.0106 (14)	0.0193 (18)	0.0220 (17)	0.0026 (12)	0.0027 (13)	0.0004 (14)
C13	0.0200 (16)	0.0176 (18)	0.0256 (18)	−0.0008 (13)	0.0012 (14)	0.0006 (14)
C14	0.0264 (18)	0.0151 (18)	0.0223 (17)	0.0008 (14)	0.0002 (14)	0.0039 (14)
C15	0.0257 (17)	0.0188 (18)	0.0179 (17)	−0.0056 (13)	0.0034 (13)	−0.0005 (14)
C16	0.0200 (16)	0.0200 (18)	0.0168 (16)	−0.0042 (13)	0.0020 (13)	−0.0029 (14)
C17	0.0212 (17)	0.0212 (19)	0.0206 (17)	−0.0029 (13)	0.0087 (14)	−0.0009 (15)
C18	0.0183 (16)	0.025 (2)	0.0217 (18)	−0.0031 (13)	0.0077 (13)	−0.0046 (15)
C19	0.0172 (16)	0.0228 (19)	0.0197 (17)	0.0000 (13)	0.0021 (13)	−0.0081 (14)
C20	0.0146 (15)	0.0243 (19)	0.0257 (18)	0.0052 (13)	0.0035 (13)	−0.0055 (15)
C21	0.0202 (16)	0.0187 (18)	0.0231 (17)	0.0024 (13)	−0.0023 (14)	−0.0014 (15)
C22	0.0214 (16)	0.0158 (17)	0.0176 (16)	−0.0023 (13)	0.0004 (13)	−0.0013 (14)
C23	0.0173 (16)	0.0182 (17)	0.0156 (16)	−0.0026 (13)	0.0019 (13)	−0.0023 (13)
C24	0.0164 (15)	0.0213 (18)	0.0137 (16)	−0.0026 (13)	0.0028 (12)	−0.0029 (13)
O4	0.0331 (14)	0.0233 (14)	0.0275 (13)	0.0057 (10)	0.0175 (11)	0.0065 (11)
O5	0.0275 (12)	0.0140 (13)	0.0250 (13)	−0.0003 (9)	0.0090 (10)	0.0021 (10)
O6	0.0421 (15)	0.0190 (14)	0.0285 (14)	0.0000 (11)	0.0208 (11)	−0.0010 (11)
O7	0.0268 (12)	0.0139 (12)	0.0234 (12)	−0.0010 (9)	0.0104 (10)	0.0009 (10)
O8	0.0330 (13)	0.0185 (13)	0.0206 (12)	−0.0011 (10)	0.0095 (10)	0.0014 (10)
C25	0.0177 (16)	0.0173 (18)	0.0208 (17)	0.0025 (13)	0.0034 (13)	−0.0038 (14)
C26	0.0178 (16)	0.0185 (18)	0.0175 (16)	−0.0006 (13)	0.0058 (13)	0.0031 (14)
C27	0.0169 (15)	0.0177 (18)	0.0219 (17)	−0.0010 (13)	0.0003 (13)	0.0034 (14)
C28	0.0187 (16)	0.0200 (19)	0.0205 (17)	−0.0007 (13)	0.0037 (13)	−0.0018 (14)
C29	0.0165 (16)	0.0218 (19)	0.0176 (16)	−0.0022 (13)	0.0050 (13)	−0.0001 (14)
C30	0.0174 (16)	0.0180 (18)	0.0187 (17)	−0.0022 (13)	0.0010 (13)	0.0010 (14)
C31	0.0150 (15)	0.0194 (18)	0.0177 (16)	−0.0018 (13)	0.0006 (13)	0.0007 (14)
O9	0.0461 (16)	0.0310 (16)	0.0361 (16)	0.0069 (12)	−0.0028 (13)	0.0026 (13)
O10	0.0349 (14)	0.0389 (16)	0.0306 (14)	0.0126 (12)	0.0102 (11)	−0.0004 (12)
O11	0.0422 (16)	0.0483 (18)	0.0229 (14)	0.0073 (12)	0.0142 (12)	0.0090 (12)
N6	0.0336 (17)	0.0225 (17)	0.0338 (18)	−0.0058 (13)	0.0053 (15)	−0.0007 (14)
O12	0.0327 (14)	0.0323 (15)	0.0280 (14)	−0.0030 (11)	0.0095 (11)	−0.0004 (12)

### Geometric parameters (Å, º)

**Table:**

Cu—O1	2.114 (2)	C8—C9	1.367 (5)
Cu—O2	2.782 (2)	C9—C10	1.408 (5)
Cu—N1	1.974 (3)	C11—C12	1.435 (5)
Cu—N2	2.082 (3)	C13—C14	1.392 (5)
Cu—N3	1.980 (3)	C14—C15	1.375 (5)
Cu—N4	2.086 (2)	C15—C16	1.416 (5)
O1—N5	1.277 (3)	C16—C17	1.436 (5)
O2—N5	1.249 (4)	C16—C24	1.391 (4)
O3—N5	1.239 (3)	C17—C18	1.363 (5)
O4—C26	1.370 (4)	C18—C19	1.439 (5)
O5—C27	1.367 (4)	C19—C20	1.408 (5)
O6—C28	1.355 (4)	C19—C23	1.408 (5)
O7—C31	1.315 (4)	C20—C21	1.363 (5)
O8—C31	1.237 (4)	C21—C22	1.404 (5)
O4—H4A	0.8200	C23—C24	1.433 (5)
O5—H5B	0.8200	C1—H1A	0.9500
O6—H6B	0.8200	C2—H2A	0.9500
O7—H7A	0.8200	C3—H3A	0.9500
O9—N6	1.251 (4)	C5—H5A	0.9500
O10—N6	1.271 (4)	C6—H6A	0.9500
O11—N6	1.233 (5)	C8—H8A	0.9500
O12—H12A	0.8200	C9—H9A	0.9500
O12—H12B	0.8200	C10—H10A	0.9500
N1—C1	1.328 (4)	C13—H13A	0.9500
N1—C12	1.373 (4)	C14—H14A	0.9500
N2—C11	1.367 (4)	C15—H15A	0.9500
N2—C10	1.326 (4)	C17—H17A	0.9500
N3—C13	1.336 (4)	C18—H18A	0.9500
N3—C24	1.368 (4)	C20—H20A	0.9500
N4—C23	1.353 (4)	C21—H21A	0.9500
N4—C22	1.335 (4)	C22—H22A	0.9500
C1—C2	1.395 (5)	C25—C30	1.408 (4)
C2—C3	1.371 (5)	C25—C26	1.383 (5)
C3—C4	1.408 (5)	C26—C27	1.389 (4)
C4—C12	1.399 (5)	C27—C28	1.397 (5)
C4—C5	1.438 (5)	C28—C29	1.384 (5)
C5—C6	1.357 (5)	C29—C30	1.390 (5)
C6—C7	1.428 (5)	C30—C31	1.478 (5)
C7—C8	1.411 (5)	C25—H25A	0.9500
C7—C11	1.402 (5)	C29—H29A	0.9500
			
Cu···O2	2.782 (2)	C9···O10^iii^	3.387 (4)
O1···O2	2.175 (3)	C10···C29^x^	3.459 (5)
O1···N1	2.801 (3)	C10···C30^x^	3.486 (5)
O1···N3	2.989 (3)	C10···C31^x^	3.411 (5)
O1···C1	3.046 (4)	C10···O8^x^	3.367 (4)
O1···O3^i^	3.146 (3)	C10···O9^iii^	3.389 (4)
O1···C18^ii^	3.356 (4)	C10···N6^iii^	3.283 (5)
O2···O12^iii^	2.960 (3)	C11···C5^iv^	3.468 (5)
O2···C5^iv^	3.295 (4)	C12···C6^iv^	3.481 (5)
O2···O1	2.175 (3)	C12···C31	3.573 (4)
O2···C13	3.254 (4)	C13···O11^iii^	3.219 (4)
O2···C6^iv^	3.322 (4)	C14···C2^iii^	3.401 (5)
O3···O5^v^	2.859 (3)	C14···O11^iii^	3.400 (4)
O3···C20^vi^	3.282 (4)	C15···C21^ii^	3.430 (5)
O3···O3^i^	3.147 (3)	C15···C20^ii^	3.423 (5)
O3···O1^i^	3.146 (3)	C16···C20^ii^	3.510 (5)
O3···O6^v^	3.219 (3)	C16···C19^ii^	3.544 (5)
O3···N5^i^	3.032 (4)	C17···C23^ii^	3.554 (5)
O3···C18^vi^	3.363 (4)	C17···O9^ii^	3.340 (5)
O4···O5	2.696 (3)	C18···O1^ii^	3.356 (4)
O4···C8^vii^	3.332 (4)	C18···C24^ii^	3.581 (5)
O4···O12^viii^	2.730 (3)	C18···O3^xiv^	3.363 (4)
O5···C20^viii^	3.346 (4)	C18···C23^ii^	3.573 (5)
O5···C1^ix^	3.338 (4)	C19···C16^ii^	3.544 (5)
O5···C2^ix^	3.363 (4)	C19···C24^ii^	3.569 (5)
O5···O4	2.696 (3)	C20···O3^xiv^	3.282 (4)
O5···O3^ix^	2.859 (3)	C20···O5^xii^	3.346 (4)
O5···O6	2.624 (3)	C20···C15^ii^	3.423 (5)
O6···O12	2.682 (3)	C20···C16^ii^	3.510 (5)
O6···O5	2.624 (3)	C21···O8	3.413 (4)
O6···O3^ix^	3.219 (3)	C21···C15^ii^	3.430 (5)
O7···C22^x^	3.369 (4)	C22···N6	3.345 (4)
O7···O8^x^	2.648 (3)	C22···O7^x^	3.369 (4)
O7···C23^x^	3.170 (3)	C22···C1	3.474 (5)
O7···N4^x^	3.128 (3)	C22···O9	3.228 (4)
O8···O8^x^	3.223 (3)	C22···O8	2.936 (3)
O8···C22	2.936 (3)	C23···C17^ii^	3.554 (5)
O8···C21	3.413 (4)	C23···O7^x^	3.170 (3)
O8···C31^x^	3.331 (4)	C23···C18^ii^	3.573 (5)
O8···C10^x^	3.367 (4)	C24···C18^ii^	3.581 (5)
O8···O7^x^	2.648 (3)	C24···C19^ii^	3.569 (5)
O9···C10^xi^	3.389 (4)	C29···O11	3.266 (5)
O9···C1	3.338 (4)	C29···C10^x^	3.459 (5)
O9···C17^ii^	3.340 (5)	C29···C9^x^	3.402 (5)
O9···C22	3.228 (4)	C30···C10^x^	3.486 (5)
O9···C9^xi^	3.166 (4)	C31···C10^x^	3.411 (5)
O10···C3^xii^	3.395 (4)	C31···C12	3.573 (4)
O10···O12	2.708 (4)	C31···O8^x^	3.331 (4)
O10···C9^xi^	3.387 (4)	C1···H22A	2.7900
O11···C13^xi^	3.219 (4)	C2···H14A^xi^	2.9900
O11···C1	3.419 (4)	C9···H29A^x^	2.9700
O11···C2	3.359 (4)	C13···H10A	2.9300
O11···O12	3.136 (3)	C14···H2A^iii^	2.7300
O11···C14^xi^	3.400 (4)	C15···H2A^iii^	3.0100
O11···C29	3.266 (5)	C17···H12A^xv^	2.9600
O12···O10	2.708 (4)	C18···H12A^xv^	2.7800
O12···O11	3.136 (3)	C22···H7A^x^	2.9600
O12···O2^xi^	2.960 (3)	C27···H14A^xi^	3.0000
O12···O4^xii^	2.730 (3)	C28···H14A^xi^	2.8700
O12···O6	2.682 (3)	C31···H7A^x^	2.7600
O1···H18A^ii^	2.6300	H1A···O1	2.7100
O1···H1A	2.7100	H1A···O9	2.5600
O2···H6A^iv^	2.7100	H2A···C15^xi^	3.0100
O2···H5A^iv^	2.6600	H2A···C14^xi^	2.7300
O2···H12B^iii^	2.6500	H2A···H14A^xi^	2.5400
O2···H12A^iii^	2.5500	H3A···H15A^xi^	2.5200
O2···H13A	2.5900	H3A···H5A	2.5900
O3···H20A^vi^	2.4400	H3A···O10^viii^	2.4800
O3···H5B^v^	2.1600	H4A···H25A	2.4000
O3···H18A^vi^	2.5500	H4A···O12^viii^	1.9200
O4···H8A^vii^	2.6200	H4A···H12A^viii^	2.2900
O5···H8A^vii^	2.5600	H4A···H12B^viii^	2.3700
O5···H20A^viii^	2.5500	H5A···H3A	2.5900
O6···H5B	2.1300	H5A···H12B^viii^	2.2800
O6···H12A	2.7800	H5A···O2^iv^	2.6600
O6···H13A^xi^	2.8900	H5B···H20A^viii^	2.4800
O7···H25A	2.4800	H5B···O6	2.1300
O8···H29A	2.4900	H5B···N5^ix^	2.8500
O8···H7A^x^	1.8400	H5B···O3^ix^	2.1600
O8···H22A	2.6900	H6A···O2^iv^	2.7100
O9···H17A^ii^	2.4900	H6A···H8A	2.5700
O9···H9A^xi^	2.5200	H6A···N5^iv^	2.9100
O9···H22A	2.8000	H6B···H12B	2.4600
O9···H1A	2.5600	H6B···O12	1.9400
O9···H15A^ii^	2.7600	H6B···H12A	2.2000
O10···H3A^xii^	2.4800	H6B···H29A	2.3500
O10···H12B	2.1300	H7A···H7A^x^	2.5600
O10···H15A^ii^	2.5600	H7A···O8^x^	1.8400
O11···H12B	2.8700	H7A···C31^x^	2.7600
O11···H29A	2.8200	H7A···C22^x^	2.9600
O11···H13A^xi^	2.6800	H8A···O5^xiii^	2.5600
O11···H22A	2.7400	H8A···H6A	2.5700
O12···H6B	1.9400	H8A···O4^xiii^	2.6200
O12···H4A^xii^	1.9200	H9A···O9^iii^	2.5200
N1···N4	3.036 (3)	H10A···N6^iii^	2.8400
N1···N5	3.298 (4)	H10A···C13	2.9300
N1···N2	2.665 (4)	H12A···C17^xvi^	2.9600
N1···C22	3.337 (4)	H12A···H6B	2.2000
N1···O1	2.801 (3)	H12A···H4A^xii^	2.2900
N1···C11	2.387 (4)	H12A···O2^xi^	2.5500
N2···N3	3.085 (4)	H12A···H18A^xvi^	2.4700
N2···C12	2.388 (4)	H12A···C18^xvi^	2.7800
N2···N1	2.665 (4)	H12A···O6	2.7800
N3···N4	2.664 (4)	H12B···N6	2.8800
N3···C10	3.415 (5)	H12B···O11	2.8700
N3···N2	3.085 (4)	H12B···O2^xi^	2.6500
N3···O1	2.989 (3)	H12B···H4A^xii^	2.3700
N3···C23	2.381 (4)	H12B···H6B	2.4600
N4···C24	2.380 (4)	H12B···H5A^xii^	2.2800
N4···N3	2.664 (4)	H12B···O10	2.1300
N4···N1	3.036 (3)	H13A···O6^iii^	2.8900
N4···O7^x^	3.128 (3)	H13A···O11^iii^	2.6800
N5···O3^i^	3.032 (4)	H13A···O2	2.5900
N6···C9^xi^	3.405 (5)	H14A···C2^iii^	2.9900
N6···C10^xi^	3.283 (5)	H14A···H2A^iii^	2.5400
N6···C22	3.345 (4)	H14A···C28^iii^	2.8700
N1···H22A	2.8800	H14A···C27^iii^	3.0000
N5···H5B^v^	2.8500	H15A···O9^ii^	2.7600
N5···H6A^iv^	2.9100	H15A···H17A	2.5700
N6···H12B	2.8800	H15A···H3A^iii^	2.5200
N6···H10A^xi^	2.8400	H15A···O10^ii^	2.5600
N6···H22A	2.7900	H17A···H15A	2.5700
C1···O9	3.338 (4)	H17A···O9^ii^	2.4900
C1···O11	3.419 (4)	H18A···H12A^xv^	2.4700
C1···C22	3.474 (5)	H18A···H20A	2.5800
C1···O5^v^	3.338 (4)	H18A···O1^ii^	2.6300
C2···C14^xi^	3.401 (5)	H18A···O3^xiv^	2.5500
C2···O5^v^	3.363 (4)	H20A···O3^xiv^	2.4400
C2···O11	3.359 (4)	H20A···H18A	2.5800
C3···C8^iv^	3.354 (5)	H20A···H5B^xii^	2.4800
C3···O10^viii^	3.395 (4)	H20A···O5^xii^	2.5500
C4···C7^iv^	3.464 (5)	H22A···O11	2.7400
C5···C7^iv^	3.538 (5)	H22A···N1	2.8800
C5···O2^iv^	3.295 (4)	H22A···N6	2.7900
C5···C11^iv^	3.468 (5)	H22A···C1	2.7900
C6···O2^iv^	3.322 (4)	H22A···O9	2.8000
C6···C12^iv^	3.481 (5)	H22A···O8	2.6900
C7···C4^iv^	3.464 (5)	H25A···O7	2.4800
C7···C5^iv^	3.538 (5)	H25A···H4A	2.4000
C8···C3^iv^	3.354 (5)	H29A···O8	2.4900
C8···O4^xiii^	3.332 (4)	H29A···O11	2.8200
C9···N6^iii^	3.405 (5)	H29A···H6B	2.3500
C9···O9^iii^	3.166 (4)	H29A···C9^x^	2.9700
C9···C29^x^	3.402 (5)		
			
O1—Cu—O2	50.52 (8)	C17—C18—C19	121.0 (3)
O1—Cu—N1	86.42 (10)	C18—C19—C20	124.1 (3)
O1—Cu—N2	132.61 (9)	C20—C19—C23	117.0 (3)
O1—Cu—N3	93.74 (10)	C18—C19—C23	118.8 (3)
O1—Cu—N4	105.34 (9)	C19—C20—C21	119.7 (3)
O2—Cu—N1	93.06 (8)	C20—C21—C22	119.6 (3)
O2—Cu—N2	84.29 (8)	N4—C22—C21	122.1 (3)
O2—Cu—N3	88.12 (8)	C19—C23—C24	119.6 (3)
O2—Cu—N4	153.32 (9)	N4—C23—C24	117.4 (3)
N1—Cu—N2	82.09 (11)	N4—C23—C19	123.0 (3)
N1—Cu—N3	178.59 (10)	N3—C24—C23	116.5 (3)
N1—Cu—N4	96.77 (10)	N3—C24—C16	122.6 (3)
N2—Cu—N3	98.81 (11)	C16—C24—C23	120.9 (3)
N2—Cu—N4	121.57 (10)	C2—C1—H1A	119.00
N3—Cu—N4	81.84 (10)	N1—C1—H1A	119.00
Cu—O1—N5	109.30 (19)	C1—C2—H2A	120.00
Cu—O2—N5	78.26 (15)	C3—C2—H2A	120.00
C26—O4—H4A	108.00	C4—C3—H3A	120.00
C27—O5—H5B	107.00	C2—C3—H3A	120.00
C28—O6—H6B	107.00	C6—C5—H5A	120.00
C31—O7—H7A	108.00	C4—C5—H5A	120.00
H12A—O12—H12B	101.00	C5—C6—H6A	119.00
C1—N1—C12	118.5 (3)	C7—C6—H6A	119.00
Cu—N1—C1	127.4 (2)	C7—C8—H8A	120.00
Cu—N1—C12	114.0 (2)	C9—C8—H8A	120.00
Cu—N2—C11	110.5 (2)	C10—C9—H9A	120.00
C10—N2—C11	117.5 (3)	C8—C9—H9A	120.00
Cu—N2—C10	132.0 (2)	N2—C10—H10A	119.00
C13—N3—C24	118.7 (3)	C9—C10—H10A	119.00
Cu—N3—C24	113.6 (2)	C14—C13—H13A	119.00
Cu—N3—C13	127.7 (2)	N3—C13—H13A	119.00
Cu—N4—C22	131.2 (2)	C15—C14—H14A	120.00
Cu—N4—C23	110.3 (2)	C13—C14—H14A	120.00
C22—N4—C23	118.4 (3)	C14—C15—H15A	121.00
O1—N5—O3	119.0 (3)	C16—C15—H15A	121.00
O2—N5—O3	122.1 (2)	C16—C17—H17A	120.00
O1—N5—O2	118.9 (2)	C18—C17—H17A	120.00
O9—N6—O11	121.8 (3)	C19—C18—H18A	120.00
O9—N6—O10	119.1 (3)	C17—C18—H18A	120.00
O10—N6—O11	119.0 (3)	C21—C20—H20A	120.00
N1—C1—C2	122.3 (3)	C19—C20—H20A	120.00
C1—C2—C3	119.6 (3)	C20—C21—H21A	120.00
C2—C3—C4	119.7 (3)	C22—C21—H21A	120.00
C5—C4—C12	118.1 (3)	N4—C22—H22A	119.00
C3—C4—C5	124.4 (3)	C21—C22—H22A	119.00
C3—C4—C12	117.4 (3)	C26—C25—C30	119.7 (3)
C4—C5—C6	120.9 (3)	O4—C26—C25	123.1 (3)
C5—C6—C7	121.7 (3)	O4—C26—C27	117.2 (3)
C6—C7—C11	118.9 (3)	C25—C26—C27	119.8 (3)
C8—C7—C11	117.1 (3)	C26—C27—C28	120.5 (3)
C6—C7—C8	124.0 (3)	O5—C27—C26	119.9 (3)
C7—C8—C9	119.3 (3)	O5—C27—C28	119.6 (3)
C8—C9—C10	119.8 (3)	C27—C28—C29	120.1 (3)
N2—C10—C9	122.7 (3)	O6—C28—C27	114.0 (3)
C7—C11—C12	119.4 (3)	O6—C28—C29	125.9 (3)
N2—C11—C7	123.7 (3)	C28—C29—C30	119.5 (3)
N2—C11—C12	116.9 (2)	C25—C30—C31	121.3 (3)
N1—C12—C11	116.4 (3)	C29—C30—C31	118.3 (3)
N1—C12—C4	122.5 (3)	C25—C30—C29	120.4 (3)
C4—C12—C11	121.1 (3)	O7—C31—C30	115.8 (2)
N3—C13—C14	121.9 (3)	O8—C31—C30	121.5 (3)
C13—C14—C15	120.2 (3)	O7—C31—O8	122.6 (3)
C14—C15—C16	118.9 (3)	C30—C25—H25A	120.00
C15—C16—C24	117.7 (3)	C26—C25—H25A	120.00
C17—C16—C24	118.8 (3)	C28—C29—H29A	120.00
C15—C16—C17	123.5 (3)	C30—C29—H29A	120.00
C16—C17—C18	121.0 (3)		
			
O2—Cu—O1—N5	10.27 (17)	C22—N4—C23—C19	0.2 (4)
N1—Cu—O1—N5	−86.7 (2)	C22—N4—C23—C24	−178.3 (3)
N2—Cu—O1—N5	−10.8 (3)	N1—C1—C2—C3	0.6 (5)
N3—Cu—O1—N5	94.7 (2)	C1—C2—C3—C4	−1.1 (5)
N4—Cu—O1—N5	177.29 (19)	C2—C3—C4—C5	−176.6 (3)
O1—Cu—O2—N5	−10.12 (16)	C2—C3—C4—C12	0.6 (5)
N1—Cu—O2—N5	72.70 (18)	C3—C4—C5—C6	177.0 (3)
N2—Cu—O2—N5	154.43 (18)	C12—C4—C5—C6	−0.1 (5)
N3—Cu—O2—N5	−106.52 (18)	C3—C4—C12—N1	0.5 (5)
N4—Cu—O2—N5	−39.0 (3)	C3—C4—C12—C11	−176.8 (3)
O1—Cu—N1—C1	−42.0 (3)	C5—C4—C12—N1	177.8 (3)
O1—Cu—N1—C12	136.0 (2)	C5—C4—C12—C11	0.6 (5)
O2—Cu—N1—C1	−92.2 (3)	C4—C5—C6—C7	−0.8 (5)
O2—Cu—N1—C12	85.9 (2)	C5—C6—C7—C8	−176.3 (3)
N2—Cu—N1—C1	−176.0 (3)	C5—C6—C7—C11	1.2 (5)
N2—Cu—N1—C12	2.1 (2)	C6—C7—C8—C9	177.2 (3)
N4—Cu—N1—C1	63.0 (3)	C11—C7—C8—C9	−0.3 (5)
N4—Cu—N1—C12	−119.0 (2)	C6—C7—C11—N2	−178.1 (3)
O1—Cu—N2—C10	98.6 (3)	C6—C7—C11—C12	−0.7 (5)
O1—Cu—N2—C11	−79.7 (2)	C8—C7—C11—N2	−0.5 (5)
O2—Cu—N2—C10	82.4 (3)	C8—C7—C11—C12	176.9 (3)
O2—Cu—N2—C11	−95.9 (2)	C7—C8—C9—C10	1.0 (5)
N1—Cu—N2—C10	176.3 (3)	C8—C9—C10—N2	−0.9 (5)
N1—Cu—N2—C11	−2.0 (2)	N2—C11—C12—N1	0.1 (4)
N3—Cu—N2—C10	−4.8 (3)	N2—C11—C12—C4	177.5 (3)
N3—Cu—N2—C11	176.9 (2)	C7—C11—C12—N1	−177.6 (3)
N4—Cu—N2—C10	−90.6 (3)	C7—C11—C12—C4	−0.2 (5)
N4—Cu—N2—C11	91.1 (2)	N3—C13—C14—C15	0.6 (5)
O1—Cu—N3—C13	−68.7 (3)	C13—C14—C15—C16	−1.8 (5)
O1—Cu—N3—C24	110.3 (2)	C14—C15—C16—C17	−177.9 (3)
O2—Cu—N3—C13	−18.4 (3)	C14—C15—C16—C24	0.7 (4)
O2—Cu—N3—C24	160.6 (2)	C15—C16—C17—C18	176.7 (3)
N2—Cu—N3—C13	65.5 (3)	C24—C16—C17—C18	−1.9 (5)
N2—Cu—N3—C24	−115.5 (2)	C15—C16—C24—N3	1.7 (4)
N4—Cu—N3—C13	−173.6 (3)	C15—C16—C24—C23	−176.2 (3)
N4—Cu—N3—C24	5.4 (2)	C17—C16—C24—N3	−179.6 (3)
O1—Cu—N4—C22	87.0 (3)	C17—C16—C24—C23	2.5 (4)
O1—Cu—N4—C23	−97.6 (2)	C16—C17—C18—C19	0.7 (6)
O2—Cu—N4—C22	109.7 (3)	C17—C18—C19—C20	−177.9 (4)
O2—Cu—N4—C23	−74.9 (3)	C17—C18—C19—C23	0.0 (5)
N1—Cu—N4—C22	−1.2 (3)	C18—C19—C20—C21	177.5 (4)
N1—Cu—N4—C23	174.3 (2)	C23—C19—C20—C21	−0.4 (5)
N2—Cu—N4—C22	−86.0 (3)	C18—C19—C23—N4	−177.9 (3)
N2—Cu—N4—C23	89.5 (2)	C18—C19—C23—C24	0.6 (5)
N3—Cu—N4—C22	178.6 (3)	C20—C19—C23—N4	0.1 (5)
N3—Cu—N4—C23	−5.9 (2)	C20—C19—C23—C24	178.6 (3)
Cu—O1—N5—O2	−20.5 (3)	C19—C20—C21—C22	0.4 (5)
Cu—O1—N5—O3	157.7 (2)	C20—C21—C22—N4	0.0 (5)
Cu—O2—N5—O1	14.9 (2)	N4—C23—C24—N3	−1.3 (4)
Cu—O2—N5—O3	−163.2 (3)	N4—C23—C24—C16	176.7 (3)
Cu—N1—C1—C2	178.4 (2)	C19—C23—C24—N3	−179.9 (3)
C12—N1—C1—C2	0.4 (5)	C19—C23—C24—C16	−1.9 (4)
Cu—N1—C12—C4	−179.2 (3)	C30—C25—C26—O4	−179.6 (3)
Cu—N1—C12—C11	−1.8 (3)	C30—C25—C26—C27	−1.0 (5)
C1—N1—C12—C4	−1.0 (5)	C26—C25—C30—C29	0.0 (5)
C1—N1—C12—C11	176.4 (3)	C26—C25—C30—C31	177.9 (3)
Cu—N2—C10—C9	−178.0 (2)	O4—C26—C27—O5	−2.2 (4)
C11—N2—C10—C9	0.2 (5)	O4—C26—C27—C28	179.8 (3)
Cu—N2—C11—C7	179.2 (3)	C25—C26—C27—O5	179.2 (3)
Cu—N2—C11—C12	1.6 (3)	C25—C26—C27—C28	1.1 (5)
C10—N2—C11—C7	0.6 (5)	O5—C27—C28—O6	1.3 (4)
C10—N2—C11—C12	−176.9 (3)	O5—C27—C28—C29	−178.3 (3)
Cu—N3—C13—C14	−179.3 (3)	C26—C27—C28—O6	179.3 (3)
C24—N3—C13—C14	1.7 (5)	C26—C27—C28—C29	−0.3 (5)
Cu—N3—C24—C16	178.0 (2)	O6—C28—C29—C30	179.8 (3)
Cu—N3—C24—C23	−4.0 (3)	C27—C28—C29—C30	−0.7 (5)
C13—N3—C24—C16	−2.9 (4)	C28—C29—C30—C25	0.8 (5)
C13—N3—C24—C23	175.1 (3)	C28—C29—C30—C31	−177.1 (3)
Cu—N4—C22—C21	174.9 (2)	C25—C30—C31—O7	−0.9 (4)
C23—N4—C22—C21	−0.3 (4)	C25—C30—C31—O8	179.4 (3)
Cu—N4—C23—C19	−175.9 (3)	C29—C30—C31—O7	177.1 (3)
Cu—N4—C23—C24	5.5 (3)	C29—C30—C31—O8	−2.7 (5)

Symmetry codes: (i) −*x*+1, −*y*+1, −*z*+1; (ii) −*x*, −*y*+1, −*z*+1; (iii) −*x*+1/2, *y*−1/2, −*z*+1/2; (iv) −*x*+1, −*y*+1, −*z*; (v) *x*+1/2, −*y*+3/2, *z*+1/2; (vi) *x*+1, *y*, *z*; (vii) −*x*+1/2, *y*+1/2, −*z*−1/2; (viii) *x*+1/2, −*y*+3/2, *z*−1/2; (ix) *x*−1/2, −*y*+3/2, *z*−1/2; (x) −*x*, −*y*+1, −*z*; (xi) −*x*+1/2, *y*+1/2, −*z*+1/2; (xii) *x*−1/2, −*y*+3/2, *z*+1/2; (xiii) −*x*+1/2, *y*−1/2, −*z*−1/2; (xiv) *x*−1, *y*, *z*; (xv) −*x*−1/2, *y*−1/2, −*z*+1/2; (xvi) −*x*−1/2, *y*+1/2, −*z*+1/2.

### Hydrogen-bond geometry (Å, º)

Cg9 is the centroid of the C16-C19/C23/C24 ring.

**Table:**

*D*—H···*A*	*D*—H	H···*A*	*D*···*A*	*D*—H···*A*
O4—H4*A*···O12^viii^	0.82	1.92	2.730 (3)	167
O5—H5*B*···O6	0.82	2.13	2.624 (3)	119
O5—H5*B*···O3^ix^	0.82	2.16	2.859 (3)	143
O6—H6*B*···O12	0.82	1.94	2.682 (3)	150
O7—H7*A*···O8^x^	0.82	1.84	2.648 (3)	170
O12—H12*A*···O2^xi^	0.82	2.55	2.960 (3)	112
O12—H12*B*···O10	0.82	2.13	2.708 (4)	128
C1—H1*A*···O9	0.95	2.56	3.338 (4)	139
C3—H3*A*···O10^viii^	0.95	2.48	3.395 (4)	161
C8—H8*A*···O5^xiii^	0.95	2.56	3.482 (4)	165
C9—H9*A*···O9^iii^	0.95	2.52	3.166 (4)	125
C13—H13*A*···O2	0.95	2.59	3.254 (4)	128
C15—H15*A*···O10^ii^	0.95	2.56	3.457 (4)	158
C17—H17*A*···O9^ii^	0.95	2.49	3.340 (5)	150
C18—H18*A*···O3^xiv^	0.95	2.55	3.363 (4)	144
C20—H20*A*···O3^xiv^	0.95	2.44	3.282 (4)	148
C20—H20*A*···O5^xii^	0.95	2.55	3.346 (4)	141
C25—H25*A*···*Cg*9^x^	0.95	2.95	3.680 (3)	135

Symmetry codes: (ii) −*x*, −*y*+1, −*z*+1; (iii) −*x*+1/2, *y*−1/2, −*z*+1/2; (viii) *x*+1/2, −*y*+3/2, *z*−1/2; (ix) *x*−1/2, −*y*+3/2, *z*−1/2; (x) −*x*, −*y*+1, −*z*; (xi) −*x*+1/2, *y*+1/2, −*z*+1/2; (xii) *x*−1/2, −*y*+3/2, *z*+1/2; (xiii) −*x*+1/2, *y*−1/2, −*z*−1/2; (xiv) *x*−1, *y*, *z*.

## References

[bb1] Addison, A. W., Rao, T. N., Reedijk, J., van Rijn, J. & Verschoor, G. C. (1984). *J. Chem. Soc. Dalton Trans.* pp. 1349–1356.

[bb2] Brown, I. D. & Altermatt, D. (1985). *Acta Cryst.* B**41**, 244–247.

[bb3] Chen, Z. M., Li, W., Yang, Y. Q., Kuang, D. Z., Feng, Y. I., Wang, J. Q. & Zhang, F. X. (2005). *J. Nat. Sci. Hunan Normal Univ.* **28**, 54–58.

[bb4] Dumur, F., Mayer, C. R., Hoang-Thi, K., Ledoux-Rak, I., Miomandre, F., Clavier, G., Dumas, E., Méallet-Renault, R., Frigoli, M., Zyss, J. & Sécheresse, F. (2009). *Inorg. Chem.* **48**, 8120–8133. 10.1021/ic900060d 19642646

[bb5] Liebau, F. (2000). *Z. Kristallogr.* **215**, 381–383.

[bb6] Ovens, J. S., Geisheimer, A. R., Bokov, A. A., Ye, Z. G. & Leznoff, D. B. (2010). *Inorg. Chem.* **49**, 9609–9616. 10.1021/ic101357y 20860369

[bb7] Oxford Diffraction (2009). *CrysAlis CCD*, *CrysAlis RED* and *CrysAlis PRO*. Oxford Diffraction Ltd, Yarnton, England.

[bb8] Seidel, R. W., Goddard, R., Hoch, C. & Oppel, I. M. (2011). *Z. Anorg. Allg. Chem.* **637**, 1545–1554.

[bb9] Selvakumar, B., Rajendiran, V., Maheswari, P. U., Stoeckli-Evans, H. & Palaniandavar, M. (2006). *J. Inorg. Biochem.* **100**, 316–330. 10.1016/j.jinorgbio.2005.11.018 16406550

[bb10] Sheldrick, G. M. (2008). *Acta Cryst.* A**64**, 112–122. 10.1107/S0108767307043930 18156677

[bb11] Spek, A. L. (2009). *Acta Cryst.* D**65**, 148–155. 10.1107/S090744490804362X PMC2631630 19171970

[bb12] Wyllie, G. R. A., Munro, O. Q., Schulz, C. E. & Scheidt, W. R. (2007). *Polyhedron*, **26**, 4664–4672. 10.1016/j.poly.2007.03.048 PMC2151737 18852825

